# Descriptive Epidemiology of the Tuberculosis Service Delivery Project Beneficiaries in Northwest Syria: 2019-2020

**DOI:** 10.3389/fpubh.2021.672114

**Published:** 2021-08-27

**Authors:** Hani Alashavi, Mahmoud Daher, Dato Chorgoliani, Molham Saflo, Mohammed Zeidan, Fedi Huseyinibrahim, Eyup Ismail, Abdul Raouf Haj Yousef, Khalil Ayat, Ekrem Elobayd, Andre Dadu, Masood Ali Shaikh

**Affiliations:** ^1^WHO Gaziantep Field Presence, Gaziantep, Turkey; ^2^Hand in Hand for Relief and Development (NGO), Aleppo, Syria; ^3^Syria Relief and Development (NGO), Aleppo, Syria; ^4^Bahar NGO, Afrin, Syria; ^5^WHO EURO, Copenhagen, Denmark

**Keywords:** tuberculosis, epidemiology, Syria, treatment, conflict

## Abstract

**Background:** Tuberculosis (TB) is a chronic communicable disease caused by the Mycobacterium tuberculosis that thrives in protracted humanitarian crises. It is an important cause of morbidity and mortality burden in the developing world. Globally, TB is the number one cause of death from any single infectious disease agent that plagued an estimated 10 million (range, 8.9–11.0 million) people in 2019 alone. The Eastern Mediterranean region comprised 8.2% of the worldwide share of TB cases in 2019.

**Methods:** in April 2019, the World Health Organization's (WHO) country office of Turkey started three TB centers in the cities of A'zaz, Idleb, and Afrin in northwest Syria, to provide the population with quality TB treatment curative services. The objectives of the project involved provision of full package of TB services in alignment with WHO TB standards and protocols. Three contractors i.e., national NGOs, were selected after a rigorous process in accordance with WHO policies. These newly established centers were equipped with the essential medical supplies, including well-functioning X-ray and microscopy laboratories run by WHO-trained medical doctors and lab technicians.

**Results:** Based on the quarterly reports submitted by the WHO partners, from the last two quarters of the year 2019, and the four quarters for the year 2020, out of 785 cases diagnosed either by clinical, laboratory, or radiological assessment, 251 cases were bacteriologically confirmed as TB cases against the backdrop of 2236 bacteriological investigations done and a weekly average of 31 sputum specimens processed. A total of 316 smear positive slides were identified during the study period, with the proportion of smear positive slides to be 14.13%. Clinical status determined after 6 months of treatment revealed that out of the 181 patients enrolled in the third quarter of 2019, 128 patients were either cured or successfully completed their TB treatment; with a treatment success rate of 70.7% and in quarter 4, 2019 those figures were respectively: 133, 82 and 61.7%.

**Conclusion:** Despite the challenging and protracted complex humanitarian situation in northwest Syria, the number of patients enrolled and the proportion who successfully completed the TB treatment is acceptable. However, these results are preliminary, as clinical outcomes were available only for the first and second cohorts of patients enrolled. Innovative solutions and flexibility in the execution and continued expansion of this promising project are imperative.

## Introduction

Tuberculosis (TB) is a chronic communicable disease caused by the Mycobacterium tuberculosis (MTB) that is a major cause of morbidity and mortality burden in the developing world (1). Globally, TB is the number one cause of death from a single infectious agent that afflicted an estimated 10 million (range, 8.9–11.0 million) people in 2019 alone (2). While there were an estimated 1.2 million deaths attributed to TB among HIV-negative people in 2019 and an additional 208 000 deaths (range, 177 000–242 000) in HIV-positives (2). A recent meta-analysis reported that globally about 1.7 billion people are infected with MTB (3). A 2020 modeling study reported that in low- and middle-income countries, risk of TB deaths in the next five years could increase by 20% owing to Covid-19 in high burden countries (4).

Regarding geographic distribution, the highest proportion of the TB afflicted population in 2019 lived in the WHO regions of South-East Asia (44%), followed by Africa (25%) and the Western Pacific (18%). Whereas the Eastern Mediterranean region comprised an 8.2% share of TB cases (2).

Disasters, whether man-made or natural, exact their greatest toll on the lowest rungs of the social ladder. Protracted crises like civil war in Syria have transmogrified the country into breeding grounds of high disease morbidity and mortality burdens, including infectious diseases like TB (5). Such complex humanitarian situations have been reported to increase TB risk by a whopping 20 fold (6). TB thrives in overcrowded living environments coupled with low sanitation conditions conducive to TB. Such conditions are ripe against the backdrop of protracted crises with forced mass displacements internally as well as to external to the country like Syria (6). The decade long Syrian crisis has resulted in the disruption and devastation of economy, health systems, and key health and other infrastructures in the country.

The estimated incidence of TB was reported as 19/100,000 for the year 2019, which is considered a low TB burden (2). However, there is a need to provide timely updates on the TB burden, so as to better inform health systems – albeit fragmented – in the country, for better informed ongoing preventive and control measures. In addition to elucidating health priorities for the non-public sector healthcare service providers, it is important to foster funding, and help the donor community to better target their health development funding decisions.

In mid-2018, the World Health Organization (WHO) field office in Gaziantep, Turkey conducted an assessment of available TB services in Northwest Syria (NWS) so as to determine gaps in provision of quality preventive and curative TB services in the war-ravaged NWS. The assessment revealed many weaknesses: non-existence of reliable data on the TB burden; no surveillance system for the drug resistant cases; no responsible entity for the existing rudimentary TB services; frequent and long TB medication stockouts, weak diagnostic services; and limited knowledge of proper TB treatment and management among clinical staff.

The objectives of this study were twofold: to provide a descriptive epidemiological profile of the TB burden in terms of diagnosis, management, treatment, and outcomes based on the recently established three TB clinical service centers operated by various national NGOs in collaboration with and monitored by WHO; and to establish a baseline against which continued clinical TB control interventions in the Syria would be monitored. The epidemiological profile and clinical aspects of the TB services were gleaned from the administrative data provided by the three implementing partners to WHO.

## Methodology

In order to provide quality TB treatment services, in April 2019 WHO established three TB centers in the cities of A'zaz, Idleb, and Afrin, ensuring the widest possible geographic coverage to the populations, including the Internally Displaced Population (IDP) camp populations. Three implementing partners (IP) were identified who aligned with WHO policies and TB management protocols. Refurbishing of three existing TB centers commenced in close consultation with Turkish health authorities to refurbish buildings by ensuring compliance with all pertinent standards, including installation of X-ray equipment, identification of isolation areas, patient flow management, and proper ventilation. The objectives of the project entailed provision of full package of TB services in alignment with WHO TB standards and protocols. Three contractors i.e. national NGOs named ‘Bahar Dernegi', ‘Hand in Hand for Aid and Development' and ‘Syria Relief and Development Foundation' were selected after a rigorous process that vetted all received projects after a request for proposal (RFP) in accordance with WHO policies.

These newly established centers were equipped with the essential medical supplies, including well-functioning X-ray and microscopy laboratories run by WHO-trained medical doctors and lab technicians. Patients from Idlib, Afrin, and Azaz cities as well as other parts of the two governorates have been referred to these centers to receive quality TB care. For many patients, this was their first time receiving such care.

Passive case detection was used until the fourth quarter of 2020, while from 2021 active case detection was introduced. The project started with passive case detection owing to the existence of a referral system where patients with presumptive TB symptoms identified at primary health care (PHC) facilities were referred to WHO's project funded TB centers. All family members and contacts of confirmed TB patients undergo investigation for TB and children under the age of 5 get Isoniazid preventive treatment. However, the number of functional PHC centers in the project catchment areas have varied widely owing to protracted humanitarian crises.

Specifically, WHO, through its three Ips, ensured provision of the TB services: rehabilitation of buildings according to infection control measures to fit requirements for TB center (7); introduction of WHO recommended Reporting and Recording (R&R) system (8); organization of supportive supervision visits by specially trained TB focal persons; Provision of Training for medical doctors and laboratory technicians; establishment of X-ray and microscopy laboratories; introduction of rapid molecular diagnostic service by installing Gene Xpert Rif; establishment of sputum samples transportation system from NWS to Antakya, Turkey; and adequate supply of first line TB treatment drugs (Rifampicin, Isoniazid, Ethambutol, and Pyrazinamide) was procured and ensured.

Also, the treatment of patients with drug susceptible TB was commenced based on WHO guidelines (9); TB patients' contact investigation system was established; Isoniazid preventive treatment was initiated for children under 5 years according to WHO guidelines (10); treatment supporters' teams were created, hailing from community members who underwent training and visited TB patients at home in order to monitor adherence to treatment; awareness of the TB disease was enhanced through health education sessions provided by trained health care workers either at the TB center or during home visits at community level; a referral system of TB suspect cases was activated from primary healthcare centers (PHC) to the three new TB centers after training of PHC medical doctors and establishment of transportation system of suspect patients to transport them; the introduction of mobile TB screening teams was started in all areas after the training of mobile screening team members in identification of TB presumptive cases for referral to TB centers; and TB/Covid-19 collaborative activities were introduced that ensure Covid-19 testing for all TB patients and vice versa to increase detection of TB cases (11, 12), which resulted in the identification of 9 cases with coinfections of both TB and Covid-19. These collaborative endeavors ensured increased yield in case detection for both infections.

The WHO field office in Gaziantep has been receiving monthly and quarterly compiled selected data points pertaining to the provision of TB care, as per contractual obligation on the part of three IPs. These data are received as an Excel file with various tabs providing various demographic and clinical information on the TB consultations provided and specified outcomes regularly.

In this study, descriptive analysis of the quarterly reports received from third quarter of the year 2019 to fourth quarter of 2020 i.e., six quarters, is provided.

The successful treatment was calculated as the sum of the number of cases cured and those who completed treatment, divided by the number of cases registered in the quarter. Patients who completed the treatment regimen but have not yet had their final sputum test result are deemed as having successfully completed the treatment.

## Results

Based on the quarterly reports submitted by the WHO partners, from the last two quarters of the year 2019, and the four quarters for the year 2020, a total of 295 people were identified as bacteriologically confirmed TB cases against the backdrop of 2,236 bacteriological investigations done and on a weekly average of 31 sputum specimens processed. A total of 316 smear positive slides were identified during the study period, with the proportion of smear positive slides to be 14.1%. Table 1 presents the three city disaggregated data, showing the highest proportion of smear positive slides to be 26% from the city/district of Idlib in the Idlib governorate of Northwest Syria. While lowest proportion i.e. 8.1% was reported from Afrin city in the Aleppo governorate.

**Table 1:** Results of bacteriological investigations, cumulative and disaggregated by cities in Northwest Syria.

	**Afrin**	**Idlib**	**Azaz**	**Total**
People with positive bacteriological result	48	133	70	251
Bacteriological investigations done	859	580	797	2236
Smear positive slides identified^*^	70	151	95	316
Weekly average sputum specimens processed^*a*^	12	8	11	31
Proportion of microscopy slides that are smear positive	8.1%	26%	11.91%	14.13%

a *Weekly average: Number of weeks for 6 quarters were calculated as 72. And weekly average nr of specimens processed 2236/72 = 31*.

* *Some patients had multiple microscopic investigations done*.

Table 2 provides the number of pulmonary TB cases confirmed by bacteriology, clinically, and the extra-pulmonary TB by each quarter, in terms of new, relapsed, and previously treated cases. Cumulatively over the course of six quarters, a total of 785 TB cases were identified, with 668 (85.1%) cases identified as new cases. The highest number of new cases were identified in the third quarter of 2019 i.e. 143 (21.4%) new cases. Whereas the lowest number of new cases were identified in the fourth quarter of 2020, during which 78 (11.7%) new cases were identified. Extrapulmonary cases in NWS mainly comprised of plural, lymphatic TB, while some cases of osteotuberculosis were also confirmed. Radiology, biopsies, and clinical assessments were the mode of identifying involvement of extrapulmonary sites. To limit the burden of data collection and reporting, breakdown by site is not available.

**Table 2:** Tuberculosis case investigation results by quarter in Northwest Syria.

	**Pulmonary TB**	**Pulmonary TB (Bacteriologically confirmed)**	**Extra Pulmonary TB (Clinically confirmed)**	***Total***
**Quarter 3 – 2019**				
New	68	18	57	*143*
Relapse	2	0	0	*2*
Previously treated (excluding relapse)	4	0	2	*6*
Previous treatment history unknown	0	21	9	*30*
*Total*	*74*	*39*	*68*	*181*
**Quarter 4 – 2019**				
New	33	23	55	*111*
Relapse	1	0	2	*3*
Previously treated (excluding relapse)	0	1	0	*1*
Previous treatment history unknown	5	6	7	*18*
*Total*	*39*	*30*	*64*	*133*
**Quarter 1 – 2020**				
New	29	16	37	*82*
Relapse	4	4	0	*8*
Previously treated (excluding relapse)	1	0	1	*2*
Previous treatment history unknown	0	1	0	*1*
*Total*	*34*	*21*	*38*	*93*
**Quarter 2 – 2020**				
New	46	31	60	*137*
Relapse	6	0	2	*8*
Previously treated (excluding relapse)	0	0	3	*3*
Previous treatment history unknown	1	1	0	*2*
*Total*	*53*	*32*	*65*	*150*
**Quarter 3 – 2020**				
New	47	20	50	*117*
Relapse	10	1	0	*11*
Previously treated (excluding relapse)	2	1	1	*4*
Previous treatment history unknown	2	1	1	*4*
*Total*	*61*	*23*	*52*	*136*
**Quarter 4 – 2020**				
New	27	14	37	*78*
Relapse	1	0	1	*2*
Previously treated (excluding relapse)	4	3	1	*8*
Previous treatment history unknown	2	0	2	*4*
*Total*	*34*	*17*	*41*	*92*

Table 3 provides the age and gender disaggregated data cumulatively, on the number of pulmonary TB confirmed cases by bacteriology, clinically, and the extra-pulmonary TB by each quarter, in terms of new, relapsed, and previously treated cases. Age was disaggregated into four groups, with the 36 years old and over as the last and the widest age group. Both genders were about equally distributed, with males comprising 410 (52.2%) cases and females comprising 375 (47.8%) cases out of the total of 785 TB cases identified. There were 39 (5%) cases in the under 5 years old age group, while there were 335 (42.7%) cases in the over 35 years old age group.

**Table 3:** Tuberculosis project beneficiaries' age and gender disaggregated information by quarter in Northwest Syria.

**Age group**	**Male**	**Female**	***Total***
**Quarter 3 – 2019**			
0 – 5 years	2	3	*5*
6 – 18 years	12	8	*20*
19 – 35	40	47	*87*
36 and over	38	31	*69*
*Total*	*92*	*89*	*181*
**Quarter 4 – 2019**			
0 – 5 years	5	1	*6*
6 – 18 years	9	6	*15*
19 – 35	23	30	*53*
36 and over	34	25	*59*
*Total*	*71*	*62*	*133*
**Quarter 1 – 2020**			
0 – 5 years	1	4	*5*
6 – 18 years	5	6	*11*
19 – 35	13	18	*31*
36 and over	31	15	*46*
*Total*	50	43	*93*
**Quarter 2 – 2020**			
0 – 5 years	5	5	*10*
6 – 18 years	5	9	*14*
19 – 35	28	35	*63*
36 and over	35	28	*63*
*Total*	*73*	*77*	*150*
**Quarter 3 – 2020**			
0–5 years	2	3	*5*
6–18 years	8	4	*12*
19–35	30	34	*64*
36 and over	32	23	*55*
*Total*	*72*	*64*	*136*
**Quarter 4 – 2020**			
0 – 5 years	2	6	*8*
6 – 18 years	2	4	*6*
19 – 35	22	13	*35*
36 and over	26	17	*43*
*Total*	*52*	*40*	*92*

It takes a follow-up and treatment over the course of six months to determine the clinical outcomes in Tuberculosis patients. Hence, treatment outcome data were available for the cohort of patients that were registered in the third and fourth quarters of 2019 i.e., in the first and second cohorts of the study. Tables 4, 5 provide for the Q3 and Q4 of 2019, the number of TB cases confirmed either bacteriology, clinically, and the extra-pulmonary TB, in terms of the number of cases registered, cured, treatment completed, treatment failed, deaths, lost to follow-ups, and the cases not evaluated. The proportions of successful treatments are also provided in these tables. The successful treatment was calculated as the sum of the number of cases cured and those who completed treatment, divided by the number of cases registered in the quarter. Patients who completed the treatment regimen but have not yet have had their final sputum test result are deemed as having successfully completed the treatment. Out of the 181 patients enrolled in the third quarter of 2019, 128 patients either were cured or successfully completed their TB treatment; with a treatment success rate of 70.7%. In patients that started treatment in Q4, 2019 out of 132 patients 82 successfully completed treatment, with treatment success rate of 62.1%.

**Table 4:** Tuberculosis treatment outcomes in cohort of patients who started treatment regimen the in third quarter of 2019.

	**Pulmonary TB (Bacteriologically Confirmed)**	**Pulmonary TB (Clinically Confirmed)**	**Extra Pulmonary TB**	**Totally enrolled in the treatment**
**Quarter 3 – 2019**				
*Cases registered*	*74*	*39*	*68*	*181*
Cured	16	0	0	17
Treatment completed	36	27	49	111
Treatment failed	0	0	0	0
Died	4	0	0	4
Lost to follow-up	14	10	17	41
Not evaluated	4	2	2	8
Treatment successful^*^	70.3%	69.2%	72.1%	70.7%

* *Sum of cured and those who completed treatment, divided by total number of cases enrolled*.

**Table 5:** Tuberculosis treatment outcomes in cohort of patients who started treatment regimen the in fourth quarter of 2019.

	**Pulmonary TB (Bacteriologically Confirmed)**	**Pulmonary TB (Clinically Confirmed)**	**Extra Pulmonary TB**	**Totally enrolled**
**Quarter 4 – 2019**				
Cases registered	39	29	64	132
Cured	8	0	0	8
Treatment completed	15	17	42	74
Treatment failed	1	0	0	1
Died	3	2	1	6
Lost to follow-up	6	10	16	32
Not evaluated	6	0	5	11
Treatment successful^*^	58.97%	58.62%	65.62%	62.1%

* *Sum of cured and those who completed treatment, divided by total number of cases enrolled*.

## Discussion

Poverty, overcrowded living conditions, marred by poor sanitation and nutritional status, against the backdrop of compromised health systems are the breeding grounds for various infectious diseases including TB. In the absence of administrative data prior to WHO taking the lead in ensuring provision of quality Tuberculosis treatment and management services in the NWS, comparable figures are not available to gauge the changes in the TB morbidity and mortality. Over the course of six quarters, a total of 316 smear positive slides were identified, with the proportion of smear positive slides to be 14.3%. The highest proportion of smear positive slides were reported from the city of Idlib (26%) and lowest proportion from Afrin city (8.1%). Cumulatively, 785 TB cases were identified, the majority of which were new cases i.e., 668 (85.1%) cases, testifying to the success of WHO initiative in terms of identifying, treating, and controlling TB.

In the first quarter of the project, establishing routines and setting up the services, and announcing the provision of diagnostic and curative services to the general public resulted in fewer TB consultations. However, subsequently these new services were well received by the communities. Reflected by the fact that the newest cases of TB were identified in the third quarter of 2019. This was followed by the lowest number of new cases being identified in the fourth quarter of 2020, during which 78 new cases were identified. Unfortunately, as Covid-19 infection significantly compromised the TB project, patients' referral to TB centers decreased which compromised case detection and decreased the number of patients. Based on our daily discussions with the project teams in NWS, we were expecting identification of many, many more cases, but Covid-19 put a dramatic damper on these expectations. As we felt the acceptance of our TB services were much welcomed and accepted by the communities we are striving to serve. Coupled with the fact that all clinical and allied staff was hired from NWS often from the very communities we are serving. The people in NWS are struggling daily to merely survive and provide for their families; seeking TB care is not on their top few priorities.

In terms of age and gender profile of TB cases, the highest burden was borne by the age group comprising of individuals aged 36 and over. Though TB patients in this age group comprised 42.7%; this proportion is still less than what has been reported in many systematic surveys, where up to two-thirds of patients fall in this age group. Secondly, our results show an almost equal ratio of both genders afflicted with TB, whereas most studies report a higher proportion of males. However, NWS is undergoing the double whammy of chronic ongoing civil strife and Covid-19, which has limited the acceptability, availability, and access to healthcare services; coupled with the limited mobility of people, and involvement of men in war fronts, this might explain the aberration in our findings.

Interestingly, the under 5 years old age group had the lowest burden of TB, as there were 39 (5%) cases in this age group. As diagnoses of TB in children is problematic owing to difficulties of sputum expectoration; in our project, TB diagnosis was based primarily on radiological findings, anamnesis, and clinical judgment of trained physicians.

Detection of bacteriologically confirmed cases are important for controlling transmission of infection. However, the humanitarian context of NWS poses its own unique challenges, including physicians' tendency to sometimes rely on radiological and clinical findings alone. This issue was highlighted during ongoing monitoring and evaluation of the project activities and addressed on an ongoing basis with refresher training emphasizing adherence to project protocols. Detection of drug resistant cases though Gene Xpert Rif commenced in the latter part of 2020's last quarter, while treatment of DRTB was subsequently introduced in February 2021. However, in this study, we only looked at data until the end of 2020.

Upon completion of the treatment regimen, TB patients did not produce sputum, which explains the increased number of those who were assigned to group ‘treatment completed'. The high number of TB patients who were lost to follow up is not uncommon in non-crisis situations. This loss to follow up is more pronounced in our project owing to the protracted humanitarian crises in NWS.

Successful TB treatment entails six-month long follow-up and, coupled with the fact that for several years no quality healthcare services were available in the region, the treatment success rate for the cohort of patients enrolled in the third quarter was high i.e. 70.3%. Which is encouraging overall; however, few were cured, and large numbers also completed treatment, while a high proportion of cases were afflicted with extrapulmonary TB. Hence, for bacteriologically confirmed cases, this is not very encouraging. The impetus for better outcomes is imperative in terms of better diagnostics and clinical management of cases. Nonetheless the context of NWS is challenging as well, with the ongoing and flux humanitarian situation in the study areas.

Hence, lower rates of successful treatment against the humanitarian backdrop are understandable, but the decline in subsequent quarters needs remedial measures that include more targeted clinical trainings.

Ten (3.2%) deaths were also reported among the cohorts of patients enrolled in the first two quarters of the study. In cohort 1 and cohort 2, treatment outcomes demonstrated relatively low successful treatment rates −70.7% and 62.1%. Those weaknesses have several explanations: challenging security situation and the necessity of 6 months duration of TB treatment, bad adherence to treatment, nature of patients who are mainly from the Internally Displaced Population, so they change living places frequently. All those factors are coupled with the fact that for several years no quality healthcare services were available in the region and doctors had no experience of managing TB cases. All this augurs for the need to further improve and expand the TB services in the region.

This descriptive study was based on administrative data collected by the project that prioritized provision of diagnostic, management, and curative services, while ensuring that there was minimal burden on data collection and reporting. As such, this administrative data is fraught with limitations in terms of the lack of more nuanced data on the burden and outcomes in TB cases. More indices were reported by WHO implementing partners in the aggregates rather than those disaggregated by demographics and geographic location of the cases.

However, plans are afoot to report more granular information in the quarterly reports from mid-2021. However, fraught political situation, unexpected and sudden population movements, and existence of a suboptimal healthcare delivery system including limited referral systems pose ongoing challenges. The world Health Organization has set the ambitious goal of reducing TB morbidity and mortality burden by 90 and 95%, respectively, from the years 2015 to 2035. Achieving this public health ambition necessitates sustained, better, and continued collaboration among all the stakeholders in the NWS owing to the challenging environment in which TB is tacked. Need for innovative interventions to address the shifting and often worsening humanitarian crises would be imperative.

To further improvise on the existing TB services, and ensure meeting the WHO TB goals, this project will prioritize on several new and innovative modalities and services including the 'Implementation of Programmatic Management of Drug Resistance TB' (PMDT), that would entail establishing a transportation system of samples from NWS to Antakya and Gaziantep, link with Gaziantep University laboratory as a referral laboratory for culturing and drugs susceptibility testing for first (FLD) and second line TB drugs (SLD); ensuring an uninterrupted supply of SLD, continued training of doctors in management of MDR patients; improving case finding of TB by strengthening mobile TB screening teams (MST); management of TB patients with co-infection of Covid-19 and/or HIV – TB/Covid-19 collaborative activities to be extended geographically in all areas of NWS; testing of TB patients on HIV to be introduced in cooperation with other NGOs working in the area; further increasing the role of PHC facilities in the TB project, and improving the quality of the referral system. The ongoing capacity building of PHC medical staff in order to ensure better integration of TB services in the PHC system. Finally, expanding the provision of TB services in NWS by adding more treatment centers, especially in places of the NWS that are difficult to access either by currently available outreach services, or patients.

In this study, our objective was to critically look at, analyze data, and to report and publish results for the first six quarters, since the inception of our TB project. We ensured rigorous enforcement and quality assurance protocols for data collection, validation, and reporting during these first six quarters, with extensive funding for data teams at all project sites. The data is and will consistently be collected in the same manner at all the project sites. However, at this juncture, we do not have adequate data team members to review all patient files, collate, compile, report, and transfer data to our Gaziantep office. The WHO office is planning to analyze this data again in 2022 i.e., for another six quarters, and report the comparisons with the data we have presented in this manuscript.

More nuanced future iterations of the descriptive analysis of this TB project would better elucidate the need for course corrections and determine impact of TB control activities in the region. Ongoing and improvised project monitoring, extending descriptive epidemiological analysis to analytical modeling of the program data would be imperative for ensuring that TB services are better aligned with the shifting ground realities of NWS.

## Conclusion

Despite trying and protracted humanitarian circumstances, the initiation, continuation, and continued expansion of quality curative services for TB treatment was made possible in northwest Syria. Clinical outcomes in the first cohort of patients are quite encouraging. However, there is a need for continued expansion of these services in terms of geographical coverage and outreach services to meet the WHO goal of reducing TB morbidity and mortality burden by 90 and 95%, respectively, from the years 2015 to 2035.

## Data Availability Statement

The raw data supporting the conclusions of this article will be made available by the authors, without undue reservation.

## Author Contributions

HA contributed to writing and revising the manuscript. MD critically reviewed the manuscript and approved for submission. DC co-wrote the first draft of the manuscript, reviewed data analysis results, and contributed to revisions. MSa, MZ, FH, EI, AY, KA, EE, and AD critically reviewed the manuscript. MSh conceived the idea, wrote the first draft of the manuscript, analyzed data, and contributed to revisions. All authors contributed to the article and approved the submitted version.

## Conflict of Interest

The authors declare that the research was conducted in the absence of any commercial or financial relationships that could be construed as a potential conflict of interest.

## Publisher's Note

All claims expressed in this article are solely those of the authors and do not necessarily represent those of their affiliated organizations, or those of the publisher, the editors and the reviewers. Any product that may be evaluated in this article, or claim that may be made by its manufacturer, is not guaranteed or endorsed by the publisher.

## References

[1] Acharya B Acharya A Ghimire SP Mishra G Parajuli N. Advances in diagnosis of Tuberculosis: an update into molecular diagnosis of Mycobacterium tuberculosis. Mol Biol Rep. (2020) 47:4065–75. 10.1007/s11033-020-05413-7 32248381

[2] World Health Organization. Global Tuberculosis Report 2020. Geneva, World Health Organization (2020).

[3] Cohen Mathiasen VD Schon T Wejse C. The global prevalence of latent tuberculosis: a systematic review and meta-analysis. Eur Respir J. (2019) 54:1900655. 10.1183/13993003.00655-2019 31221810

[4] Hogan AB Jewell BL Sherrard-Smith E Vesga JF Watson OJ Whittaker C. Potential impact of the COVID-19 pandemic on HIV, tuberculosis, and malaria in low-income and middle-income countries: a modelling study. Lancet Global Health. (2020) 8:e1132–41. 10.1016/S2214-109X(20)30288-6 32673577 PMC7357988

[5] Kimbrough W Saliba V Dahab M Haskew C Checchi F. The burden of tuberculosis in crisis-affected populations: a systematic review. Lancet Infect Dis. (2012) 12:950–65. 10.1016/S1473-3099(12)70225-6 23174381

[6] Kimbrough W Saliba V Dahab M Haskew C Checchi F. The burden of tuberculosis in crisis-affected populations: a systematic review. Lancet Infect Dis. (2012) 12:950–65. 23174381 10.1016/S1473-3099(12)70225-6

[7] WHO Guidelines on tuberculosis infection prevention and control. 2019 update (WHO/CDS/TB/2019.1). Geneva, World Health Organization. (2019). Available online at: https://apps.who.int/iris/bitstream/handle/10665/311259/9789241550512-eng.pdf 30933444

[8] Definitions and Reporting framework for tuberculosis. revision 2013 (updated version 2014) - WHO, (2014)

[9] Guidelines for treatment of drug susceptible tuberculosis and patient care. 2017 update – WHO (2017)

[10] WHO operational handbook on Tuberculosis, module 1; tuberculosis preventive treatment – WHO (2020)

[11] World Health Organization advice for the public. Available online at: https://www.who.int/emergencies/diseases/novel-coronavirus-2019/advice-for-public

[12] World Health Organization (WHO) Information Note Tuberculosis and COVID-19. COVID-Considerations for Tuberculosis (TB) Care Services. WHO (2020).

